# Efficacy and Safety of Transcutaneous Acupoint Electrical Stimulation in the Treatment of Cervical Spondylosis: A Multicenter Randomized Open‐Labeled Controlled Clinical Study

**DOI:** 10.1155/prm/3949302

**Published:** 2025-12-05

**Authors:** Lele Huang, Wenjuan Song, Xiaowei Luan, Lei Shi, Shuangyue Li, Mengcheng Cai, Jiahao Du, Ping Shi, Fanfu Fang

**Affiliations:** ^1^ Department of Rehabilitation Medicine, The First Affiliated Hospital of the Naval Medical University, Shanghai, China; ^2^ School of Health Science and Engineering, University of Shanghai for Science and Technology, Shanghai, China, usst.edu.cn; ^3^ Shanghai 411 Hospital, China Rong Tong Medical Healthcare Group Co. Ltd., Shanghai, China; ^4^ Department of Rehabilitation Medicine, Yueyang Hospital of Integrated Traditional Chinese and Western Medicine, Shanghai University of Traditional Chinese Medicine, Shanghai, China, shutcm.edu.cn; ^5^ Department of Rehabilitation Medicine, PLA Naval Medical Center, Shanghai, China

**Keywords:** cervical spondylosis, integrative medicine, neck pain, transcutaneous electrical nerve stimulation, wrist–ankle acupuncture

## Abstract

**Objective:**

To evaluate and compare the safety and efficacy of two treatments for cervical spondylosis. One treatment involves the use of electronic wrist–ankle acupuncture, in combination with Tuina, while the other treatment involves using traditional wrist–ankle acupuncture, along with Tuina. The objective was to provide evidence‐based support for the treatment of cervical spondylosis using innovative medical device E‐WAA combined with Tuina and provide a better treatment option for patients with cervical spondylosis.

**Method:**

A total of 164 patients with cervical spondylosis from June 2021 to December 2022 were randomly assigned to a control group and an experimental group. The control group received traditional wrist–ankle acupuncture combined with Tuina, while the experimental group received the electronic wrist–ankle acupuncture combined with Tuina. Both groups underwent treatment twice a week for a total of 3 weeks. The primary outcome measure was the improvement rate of visual analog scale score, while the secondary outcome measures were the Neck Disability Index score and cervical range of motion. Safety measures included the recording of adverse reactions during treatment and subjective evaluation of the treatment methods by the patients. Clinical data were analyzed from January to March 2023.

**Result:**

Both treatment modalities demonstrated improvements in pain and cervical function. There was no significant difference in the VAS improvement rate between the two groups (*p* = 0.593). However, the electronic wrist–ankle acupuncture combined with Tuina showed an additional benefit in reducing the NDI scores compared to the control group (*p* = 0.017). During the study, no adverse reaction was reported, and 83.75% of patients considered the electronic wrist–ankle acupuncture device to be safe.

**Conclusion:**

Both the electronic wrist–ankle acupuncture combined with Tuina and the traditional wrist–ankle acupuncture combined with Tuina showed significant therapeutic effects on cervical spondylosis. In comparison with the traditional wrist–ankle acupuncture, the electronic wrist–ankle acupuncture offers advantages such as noninvasiveness, controllability, and autonomous operation.

**Significance:**

These findings suggest that the electronic wrist–ankle acupuncture can serve as an adjunctive therapeutic approach for patients with cervical spondylosis, providing a more convenient treatment option.

**Trial Registration:**

Clinical Trial Registry: ChiCTR2200056394.

## 1. Introduction

Cervical spondylosis, a prevalent degenerative musculoskeletal disease, primarily manifests with neck pain [1]. This discomfort significantly impairs daily activities, compromises physical health, and diminishes work productivity [2]. Furthermore, it adversely affects sleep quality and may even impact mental and psychological well‐being. Several factors, including prolonged poor posture, aging, occupation, and trauma, contribute to the onset of cervical spondylosis [3]. The prevalence of cervical spondylosis in special occupational groups has been increasing year by year and has even reached 46.3% in some countries [4]. Due to the lack of specific treatment for cervical spondylosis, the extensive use of pain‐relieving medications has resulted in significant economic burdens on individuals, families, and society [5]. Cervical spondylosis has become a serious public health issue.

Patients favor nonpharmacological treatments due to their safety, convenience, effectiveness, and minimal side effects [6]. Tuina, a traditional Chinese medical therapy renowned for its safety, efficacy, and rapid action [7], is frequently combined with acupuncture to improve the efficacy of treatment. Tuina, as a nonpharmacological therapy, can be used to alleviate the symptoms of cervical spondylosis and is classified as Class A Level of Evidence in *Clinical Practice Guidelines for Rehabilitation of Traditional Chinese Medicine*, with proven efficacy [8, 9]. Modern studies have shown that Tuina modulates mechanosensitive channel proteins and reduces peripheral inflammatory responses [10, 11], thus relieving neck pain and improving cervical joint mobility [8]. However, Tuina’s efficacy is often short‐lived, necessitating its combination with wrist–ankle acupuncture (WAA) in clinical practice to prolong its benefits through continuous stimulation. WAA, a traditional Chinese acupuncture technique, enjoys widespread acceptance among patients with cervical spondylosis [12]. Previous investigations have indicated that acupuncture effectively alleviates neck pain compared to sham acupuncture, with effects lasting up to 3 months [13].

WAA therapy was developed by Shanghai Changhai Hospital [14], which has evolved as a separate subdiscipline within the field of acupuncture [15]. Renowned for its simplicity and high safety profile, WAA therapy is widely utilized in clinical pain management. It has shown significant therapeutic effects on cancer pain, shoulder pain, neck pain due to cervical spondylosis, primary dysmenorrhea, and postoperative pain [16–19]. Traditional WAA primarily relies on needle puncture, a technique that patients cannot self‐administer or adjust stimulation intensity. Moreover, being an invasive therapy, traditional WAA carries the risk of infection.

In contrast, the electronic WAA (E‐WAA) analgesic device integrates principles from traditional WAA and transcutaneous electrical nerve stimulation (TENS) from modern medicine. This device not only provides noninvasive TENS treatment but also maximizes the analgesic benefits of WAA [20]. Additionally, it enables patients to control stimulation intensity themselves, reducing the workload for healthcare providers and easing financial burdens for patients [21]. Clinical studies have demonstrated the efficacy of E‐WAA in improving neck pain and discomfort [22].

At present, traditional WAA combined with Tuina has been widely used in the clinical treatment of cervical spondylosis. However, due to the limitations of traditional Chinese medicine research methodology, the evidence for the clinical efficacy of combined application of WAA and Tuina is limited. The E‐WAA is noninvasive, simple, and adjustable compared with traditional WAA. Therefore, the objective of this study was to conduct an open‐label, randomized clinical trial to compare the efficacy of the E‐WAA combined with Tuina with that of traditional WAA combined with Tuina in the treatment of cervical spondylosis, so as to provide a supporting evidence for the application of the E‐WAA combined with Tuina for managing cervical spondylosis and to provide enhanced treatment alternatives for patients suffering from this condition.

## 2. Method

### 2.1. Study Design

A two‐center, open‐label, analytically blinded, randomized clinical trial was conducted from June 2021 to December 2022 at the Departments of Rehabilitation Medicine of Yueyang Hospital of Integrated Traditional Chinese and Western Medicine of Shanghai University of Traditional Chinese Medicine (2022‐028) and Changhai Hospital of Naval Medical University (2023‐007), with a total of 164 patients with cervical spondylosis enrolled, all of whom signed informed consent forms. The study was conducted in accordance with the principles of the Declaration of Helsinki and was approved by the Ethics Committees of Yueyang Hospital of Integrative Medicine of Shanghai University of Traditional Chinese Medicine and Changhai Hospital of Naval Medical University.

### 2.2. Eligibility Criteria

Patients diagnosed as cervical spondylosis were included in this study. The inclusion criteria were as follows: 18–70 years old, no gender restriction; meeting the diagnostic criteria of cervical or radicular cervical spondylosis, with pain symptoms and pain visual analog scale (VAS) score ≥ 4, without surgical indication; not taking pain medication within two weeks prior to enrollment; and voluntarily participating in the study and signing the informed consent form. The exclusion criteria were as follows: those with neck pain caused by other diseases; those with other serious diseases such as tumor, tuberculosis, and ankylosing spondylitis; combined with serious cardiovascular, cerebrovascular, hepatic, renal, or other serious life‐threatening primary diseases; those with cognitive, psychiatric, or communication dysfunctions; pregnant and breastfeeding women; those with localized skin breakage; those who were unsuitable for needling and Tuina; those who had taken part in other clinical trials on the month prior to enrollment or were taking part in other trials; those who did not perform rehabilitation therapy as specified in the study protocol; those who had poor compliance during the study, which affected the evaluation of effectiveness and safety; those who had serious adverse events, complications, or special physiological changes that made them inappropriate to accept the continuation of the study; or those who withdrew before the end of the course of treatment, lost their visits, or died due to a variety of other reasons.

### 2.3. Randomization, Allocation Concealment, and Blinding

This study was a two‐center, open‐label, analytically blinded, randomized controlled, noninferiority clinical trial. Patients were randomly divided into an experimental group and a control group in a ratio of 1:1 using a random number table generated by SPSS 26.0 software. The randomized group numbers were sealed in light‐proof envelopes. When the subjects were enrolled, they opened the envelope and were assigned to the corresponding group.

### 2.4. Interventions

The doctors treated the patients in the two groups with Tuina for cervical spine as a conventional basic therapy, using the root of the palm of both hands to push and knead following the direction of the trapezius, latissimus dorsi, and sacrospinous fibers with moderate force for 3 minutes. Additionally, the doctor kneaded the sides of the neck with the thumb and other four fingers, applying even force for 5 minutes, used the left thumb to push against the patient’s cervical spinous processes or transverse processes while holding the patient’s chin with the right hand, and guided the patient to relax the cervical region, lower the head, and flex the neck by 15°–30°. Then, the doctor slowly rotated the patient’s head to the right, following the bending of their right hand. When reaching the maximum limit with resistance, the doctor quickly pulled to the right, pushing with his/her left thumb. This action needs to be coordinated and may be accompanied by a clicking sound. The patient can be seated or in a prone position, while the doctor lightly taped or struck the back of the neck and scapular region by a clenched fist or hollow palm applying moderate strength for three to five times.

Traditional WAA treatment was adopted on the basis of conventional Tuina treatment. According to *Wrist and Ankle Acupuncture* [14], the Upper 5 zone (located in the middle between the two most prominent palmaris longus tendons and the radial flexor carpi radialis tendon on the surface of the palm of the hand) or the Upper 6 zone (1 cm from the ulnar margin of the lateral side of the little finger) was selected according to the location of the patient’s cervical spine pressure pain point, and the treatment area was sterilized by the operator. A sterile 0.25‐mm × 25‐mm acupuncture needle was used. The operator fixed the lower part of the needling point with one hand, and the thumb, index finger, and middle finger of the other hand grasped the handle of the needle and punctured the patient’s superficial subcutaneous tissues at 30° to feel the looseness during insertion, and the patient did not have any sourness, numbness, distension, or pain during the procedure, and the needle naturally lay down close to the surface of the skin. After the needling was completed, a sterile dressing was given to cover the puncture point, and a medical tape was used to fix the handle of the needle, and the needle was left in place for 30 min, and Tuina was applied at the same time. The treatment was given twice a week for a total of six sessions.

**Figure 1:**
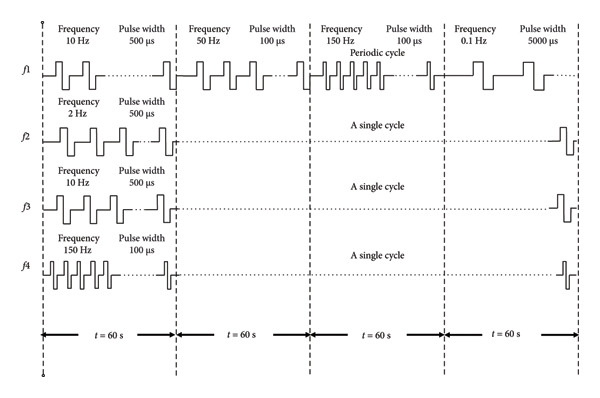
Schematic diagram of the amplitude and frequency of E‐WAA.

For the E‐WAA treatment, the treatment was carried out using the “Wearable E‐WAA Analgesia Device” developed by our research group. Figure 1 is a schematic diagram of the amplitude and frequency of E‐WAA. A low‐frequency pulse electrical stimulation primarily promotes the release of beta‐endorphins and enkephalins, while a high‐frequency pulse electrical stimulation mainly increases the release of dynorphins [23, 24]. The mixed waveform is more effective for pain relief compared to a single therapeutic effect; therefore, this study employed the *f*1 waveform mode [25].

**Figure 2:**
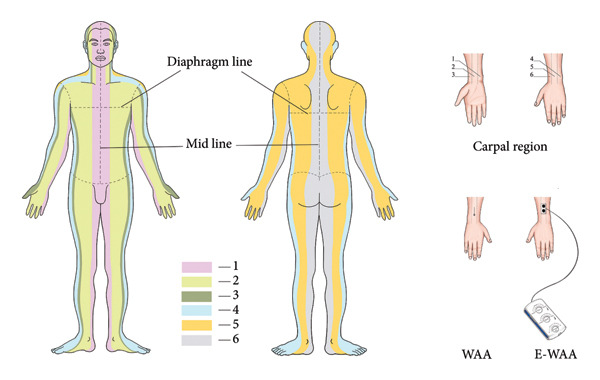
Body regions and positioning.

The patient’s treatment area was exposed and sterilized before treatment. Two transcutaneous electrical stimulation probes (electrode sheets) were placed in the Upper 5 or Upper 6 zone of WAA at the same time, with the probes close to the skin, and the appropriate pulse parameters were chosen, symmetrical bidirectional sparse‐dense wave, comfortable intensity, voltage 30–50 V, frequency 0.1–150 Hz, wave width 100–5000 μs, and a stimulation duration of 30 min. During the stimulation, Tuina was applied. The treatment was given twice a week for 3 weeks with six times in total. The partitioning and positioning of WAA and E‐WAA are shown in Figure 2. The point selection principle of WAA is that the acupuncture site is in the wrist for diseases above the diaphragm. For diseases below the diaphragm, the acupuncture site is in the ankle, and the tip is toward the lesion site. The WAA is divided into six areas: Areas 1, 2, and 3 on the front of the body, and Areas 4, 5, and 6 on the back of the body. The selected points of WAA for cervical spondylosis were in Upper 5 and Upper 6 regions. Therefore, in this study, WAA and E‐WAA were used to select the wrist acupuncture points.

### 2.5. Outcomes

The eligible patients were randomly divided into two groups, and the patients were evaluated before treatment, after three treatments, and after six treatments.

As VAS exhibits good reliability and validity in the assessment of neck pain [26], the main outcome of this study was the rate of improvement of VAS and the observation of pain relief. Referring to the clinical efficacy evaluation criteria of *Diagnostic Efficacy Criteria for Chinese Medicine Diseases* and the VAS score improvement rate for efficacy evaluation [27], the VAS score improvement rate was calculated as [(pretreatment VAS score) − (post‐treatment VAS score)/pretreatment VAS score]  × 100%. Cure: complete disappearance of symptoms, no local pain, recovery of cervical spine function, and a VAS score of zero. Significantly effective: obvious relief of symptoms, obvious reduction of local pain, obvious improvement of cervical spine function, and improvement rate of VAS score > 60%. Effective: improvement of symptoms, reduction of local pain, relief of cervical spine function, and improvement rate of VAS score of 30%–60%. Ineffective: no improvement of symptoms and improvement rate of VAS score < 30%.

The secondary outcome measures included the neck disability index (NDI) score and cervical joint mobility. The NDI is a commonly used scale for assessing the degree of functional impairment in patients with cervical spine disease [28]. The scale contains 10 items that cover various activities related to the cervical spine in daily life [29]. Each item has six levels ranging from 0 to 5, corresponding to no difficulty, mild difficulty, moderate difficulty, severe difficulty, inability to complete the activity, and inability to complete any activity. The total score ranges from 0 to 50, with higher scores indicating more severe functional impairment. In this study, the NDI score was used to assess functional impairment and quality of life in patients with cervical spondylosis, which can quantitatively assess the degree of difficulty encountered by the patients in performing various daily activities, such as neck range of motion, work ability, sleep, personal care, and household chores.

Cervical joint mobility was measured using a joint mobility scale. Prior to measurement, subjects were asked to sit up straight or stand and ensure that their head and body were in a stable position. The range of motion of the cervical spine in all directions was measured using an arthromobility scale according to the specific measurement position. Measurements included flexion, extension, left and right lateral flexion, and left and right rotation of the cervical spine. Following treatment completion, subjects in the experimental group rated the safety, convenience, comfort, and stability of the device using a Likert rating scale [30]. Before commencing the experiment, the medical staff involved in the trial was trained to standardize the measurement methods and treatment techniques to ensure data consistency.

### 2.6. Statistical Analysis

According to previous clinical studies, the WAA group (*n* = 30) had an effective rate of 86.0% after the intervention. The transcutaneous electrical stimulation group (*n* = 30) had an effective rate of 83.3% after intervention [31]. The sample size formula for this trial is as follows [32]:

(1) n=2π1−πμ1−α+μ1−βδ2,

where *n* is the sample size, *α* is the test level (*α* = 0.025) (one‐sided), *β* is the Type II error (*β* = 0.2), *δ* is the noninferiority cutoff set at 0.2, the ratio of the control group to experimental group is 1:1, and the dropout rate is 20%. Each group requires 82 patients, making a total of 164 patients needed.

SPSS 26.0 software was used for statistical analysis. Graphs were drawn using GraphPad Prism 9.3. Measurement data were first tested for normality. Data that followed a normal distribution were described using mean ± standard deviation (Mean ± SD). Data that did not conform to a normal distribution were described using median. Count data were presented as constitutive ratios. Comparisons within the groups of VAS, NDI, and CROM were made by repeated‐measures analysis of variance (ANOVA), and comparisons between the groups were made by one‐way ANOVA. For count data, the chi‐square test was used for the component ratios. Data were analyzed based on the principle of intention‐to‐treat analysis, and all patients were included in the analysis. Two‐sided tests were performed, and a difference of *p* < 0.05 was considered statistically significant. Missing values were replaced by serial means.

## 3. Results

### 3.1. Participant Characteristics

**Figure 3:**
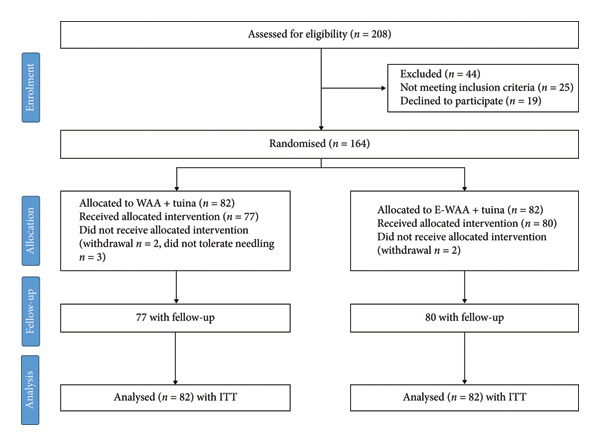
Flowchart of trial (WAA: wrist–ankle acupuncture; E‐WAA: electronic wrist–ankle acupuncture; ITT: intention to treat).

A total of 164 patients were eligible for enrollment, who were randomly assigned into a control group and experimental group, with 82 patients in each group. A total of seven cases of dropout occurred in the experiment, which was lower than the dropout rate set during the experimental design. The differences of age, height, weight, disease duration, VAS, NDI, and joint mobility between the control group and the experimental group before treatment were not statistically significant, as shown in Table 1. A total of seven cases were dislodged, which was lower than the dislodgement rate set in the experimental design. The flow chart is shown in Figure 3 [33].

**Table 1:** Characteristic data based on ITT.

Characteristic	WAA combined with Tuina	E‐WAA combined with Tuina	*p*
Age, median (SD), y	39.13 (11.58)	37.79 (10.66)	0.441
Sex			0.200
Male, %	35 (42.70)	27 (32.90)	
Female, %	47 (57.30)	55 (67.10)	
Height, median (SD), cm	166.70 (8.49)	167.34 (7.82)	0.613
Weight, median (SD), kg	64.71 (11.42)	62.45 (10.80)	0.194
Duration, median (SD), month	41.97 (54.70)	42.69 (46.85)	0.928
VAS score, median (SD)	5.97 (0.98)	5.96 (1.09)	0.943
NDI score, median (SD)	32.97 (7.53)	31.19 (5.59)	0.086
Active range of motion, median (SD), degrees			
Flexion	39.56 (6.04)	38.75 (6.23)	0.396
Extension	36.23 (8.34)	38.19 (7.41)	0.113
Left lateral flexion	26.61 (6.35)	25.92 (6.87)	0.148
Right lateral flexion	26.61 (6.35)	25.92 (6.87)	0.502
Left rotation	55.49 (13.20)	55.01 (13.01)	0.816
Right rotation	56.83 (13.25)	56.32 (12.17)	0.795

### 3.2. VAS Improvement Rate

**Figure 4:**
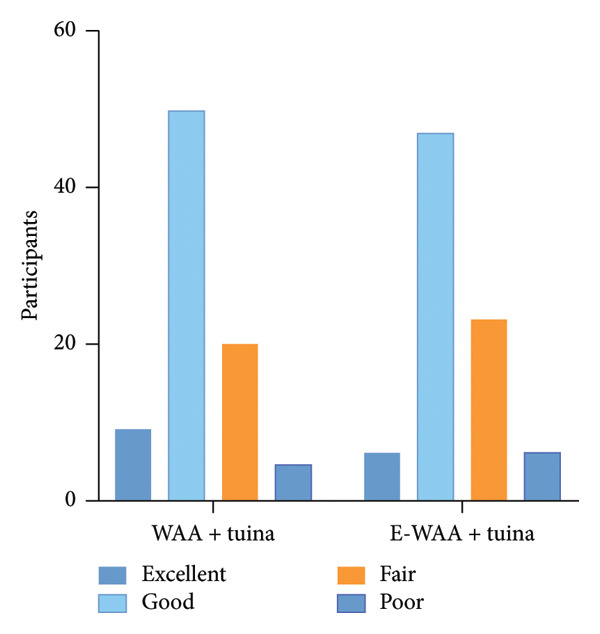
Improvement rate of VAS in the two groups.

In the control group, the efficiency was 96.3%, and there were three cases of ineffectiveness. In the experimental group, the effective rate was 92.7%, and there were six invalid cases. After the chi‐square test, there was no statistically significant difference between the two groups, indicating that the efficacies of the two groups were comparable, as shown in Figure 4. The VAS scores of both patient groups showed a significant decrease from baseline at both the 1‐week and 2‐week follow‐ups (*p* < 0.001). From a clinical significance perspective, the mean reduction in VAS scores was approximately 2.2 points at 1 week and 3.8 points at 2 weeks for both groups, exceeding the upper limit (2.5 points) of the reported minimal clinically important difference for neck pain VAS [34]. This indicates that the observed pain relief is not only statistically significant but also possesses definite clinical importance.

### 3.3. NDI and CROM

After one‐way ANOVA and post hoc pairwise comparisons, no statistical difference was observed between the two groups for the comparison of NDI and CROM before treatment, and after three and six treatments (*p* > 0.05), as shown in Table 2.

**Table 2:** Secondary outcome analysis.

Outcome	Mean (SD)		WAA combined with Tuina vs E‐WAA combined with Tuina		Group × time interaction	Time	Group
	WAA combined with Tuina	E‐WAA combined with Tuina	Difference (95% CI)	*p* value			
Primary outcome							
VAS					*F* = 0.62 *p* = 0.514	*F* = 994.17 *p* < 0.001^∗∗∗^	*F* = 0.16 *p* = 0.687
Baseline	5.97 ± 0.98	5.96 ± 1.09	0.01 (−0.31 to 0.33)	0.94			
1 week	3.75 ± 0.97	3.76 ± 1.09	−0.09 (−0.33 to 0.31)	0.95			
2 weeks	2.00 ± 1.18	2.17 ± 1.164	−0.17 (−0.53 to 0.20)	0.36			
Secondary outcome							
NDI					*F* = 0.27 *p* = 0.720	*F* = 638.61 *p* < 0.001^∗∗∗^	*F* = 5.79 *p* = 0.017^∗^
Baseline	32.97 ± 7.53	31.19 ± 5.59	1.78 (−0.26 to 3.83)	0.09			
1 week	23.84 ± 6.79	22.00 ± 5.62	1.84 (−0.08 to 3.77)	0.06			
2 weeks	15.66 ± 6.06	13.23 ± 7.15	2.44 (0.39–4.48)	0.02			
Flexion, degree					*F* = 1.42 *p* = 0.243	*F* = 134.85 *p* < 0.001^∗∗∗^	*F* = 1.05 *p* = 0.307
Baseline	39.56 ± 6.04	38.75 ± 6.23	0.82 (−1.08 to 2.70)	0.40			
1 week	41.33 ± 6.11	40.80 ± 5.54	0.53 (−1.26 to 2.33)	0.56			
2 weeks	43.90 ± 5.86	42.54 ± 5.73	1.35 (−0.43 to 3.14)	0.14			
Extension, degree					*F* = 1.27 *p* = 0.279	*F* = 163.77 *p* < 0.001^∗∗∗^	*F* = 1.90 *p* = 0.171
Baseline	36.23 ± 8.34	38.19 ± 7.41	−1.96 (−4.4 to 0.47)	0.11			
1 week	37.91 ± 6.88	38.97 ± 6.01	−0.11 (−3.05 to 0.93)	0.30			
2 weeks	41.85 ± 6.56	43.06 ± 6.09	−1.21 (−3.16 to 0.74)	0.22			
Left lateral flexion, degree					*F* = 0.11 *p* = 0.851	*F* = 336.55 *p* < 0.001^∗∗∗^	*F* = 2.80 *p* = 0.096
Baseline	26.10 ± 6.51	24.57 ± 6.93	1.53 (−0.55 to 3.60)	0.15			
1 week	30.36 ± 5.63	28.90 ± 6.75	1.45 (−0.47 to 3.36)	0.14			
2 weeks	36.54 ± 6.22	34.73 ± 8.36	1.81 (−0.46 to 4.08)	0.12			
Right lateral flexion, degree					*F* = 0.41 *p* = 0.606	*F* = 368.04 *p* < 0.001^∗∗∗^	*F* = 1.36 *p* = 0.245
Baseline	26.61 ± 6.35	25.92 ± 6.87	0.70 (−1.34 to 2.74)	0.50			
1 week	30.87 ± 5.52	29.83 ± 6.09	1.04 (−0.75 to 2.83)	0.25			
2 weeks	36.76 ± 5.59	35.40 ± 7.02	1.36 (−0.60 to 3.31)	0.17			
Left rotation, degree					*F* = 0.35 *p* = 0.615	*F* = 297.35 *p* < 0.001^∗∗∗^	*F* = 0.15 *p* = 0.701
Baseline	55.49 ± 13.20	55.01 ± 13.01	0.48 (−3.57 to 4.52)	0.82			
1 week	61.77 ± 14.70	60.59 ± 15.69	1.17 (−3.52 to 5.86)	0.62			
2 weeks	66.02 ± 14.91	65.06 ± 16.66	0.95 (−3.92 to 5.83)	0.70			
Right rotation, degree					*F* = 0.16 *p* = 0.771	*F* = 424.14 *p* < 0.001^∗∗∗^	*F* = 0.08 *p* = 0.781
Baseline	56.83 ± 13.25	56.32 ± 12.17	0.52 (−3.40 to 4.44)	0.79			
1 week	61.83 ± 14.55	61.32 ± 15.07	0.51 (−4.05 to 5.08)	0.82			
2 weeks	55.09 ± 14.84	65.27 ± 16.21	0.82 (−3.97 to 5.61)	0.74			

One asterisk (∗) indicates *p* < 0.05, statistically significant at the 0.05 level.

Two asterisks (∗∗) indicate *p* < 0.01, statistically significant at the 0.01 level.

Three asterisks (∗∗∗) indicate *p* < 0.001, highly significant.

The treatment factor had a statistically significant effect on the NDI scores, and the E‐WAA group had a better treatment effect than the WAA group. The time factor had a statistically significant effect on the decrease of NDI scores (*p* < 0.001). The post hoc pairwise comparison showed that the baseline NDI scores were significantly lower than those after three treatments and after six treatments (*p* < 0.001), and NDI scores after six treatments were significantly lower than that after three treatments (*p* < 0.001). There was no statistically significant interaction between treatment modality and assessment time (*p* > 0.05).

The treatment factor had no statistically significant effect on the increase of joint mobility, and there was no difference in the treatment effect between the WAA and E‐WAA groups. The time factor had a statistically significant effect on the increase of joint mobility (*p* < 0.001). The post hoc pairwise comparison showed that joint mobility both after three treatments and after six treatments was elevated and statistically significantly higher than that of baseline (both *p* < 0.001), and joint mobility after six treatments was significantly higher than that after three treatments (*p* < 0.001). There was no statistically significant interaction between treatment modality and assessment time (*p* > 0.05).

## 4. Discussion

After treatment, the VAS scores and NDI of cervical spondylosis patients in both groups showed a decreasing trend, while the CROM increased. There was no significant difference between the results of the two groups, indicating that WAA combined with Tuina and E‐WAA combined with Tuina both were effective in relieving cervical vertebral pain, reducing the degree of cervical vertebral dysfunction, and increasing joint mobility.

No adverse reactions occurred in the test group. A total of 83.75% of the patients commented that the E‐WAA device was safe.

Acupuncture and Tuina, as traditional modalities within Chinese medicine, are frequently employed in the management of cervical spondylosis. These interventions demonstrate efficacy in ameliorating cervical pain, optimizing blood flow, and reducing muscular tension, thereby contributing to an overall enhancement in the patient’s quality of life. Zheng et al. showed that acupuncture can relieve pain and improve long‐term well‐being in subjects with fibromyalgia [35]. A randomized controlled study published in JAMA Network Open in 2022 showed that acupuncture can be used as a safe and effective treatment for pain after Cesarean section compared to nonpenetrating placebo acupuncture [36]. In addition, U.S. government agencies are providing support for acupuncture in the treatment of acute and chronic pain, as it can also help prevent substance‐use disorders (SUDs) and drug addiction [37]. Although there are numerous studies on acupuncture for neck pain, there is still a lack of high‐quality clinical studies to verify its efficacy [38].

Since no previous studies have explored the combined use of the E‐WAA with Tuina for treating cervical spondylosis, this study may provide a new idea for complementary therapies for cervical spondylosis. We have verified the safety and effectiveness of combining the E‐WAA with the Tuina in a randomized controlled trial. Although there was no statistically significant difference in VAS scores between the two groups, patient compliance was notably higher in the experimental group compared to the control group. This may be attributed to the noninvasive feature of the E‐WAA, which reduces the likelihood of adverse reactions such as pain, swelling, and postacupuncture infections. The device’s portability, simplicity, and patient‐controlled adjustability streamline the treatment process, alleviate the workload of medical professionals, and enhance treatment efficiency. Although the therapeutic efficiency of WAA combined with Tuina was slightly higher than that of the E‐WAA combined with Tuina, this difference was primarily based on subjective patient feedback.

The E‐WAA also demonstrated additional benefits, particularly in the improvement of NDI scores. One key finding of this study was the improvement in NDI scores in the E‐WAA group, which warrants further exploration. The NDI reflects a comprehensive assessment of neck pain and related disability. The improvement observed in the experimental group may be attributed to the ability of the E‐WAA device to modulate pain through its unique electrical stimulation mechanism. Unlike traditional TENS, the E‐WAA incorporates an adaptive feature that self‐adjusts the intensity of electrical stimulation based on skin impedance, which may optimize pain relief by delivering more precise and effective therapy. This dynamic adjustment likely contributed to a more tailored and individualized approach, enhancing therapeutic outcomes.

Modern studies have shown that WAA increases the downstream pain modulation system by inhibiting the expression of 5‐hydroxytryotamine (5‐HT) and 5‐hydroxytryotamine type 3A receptor (5‐HT3AR), and the *μ*‐opioid receptor (MOR) in the medullary cephalic ventral–lateral–spinal cord pathway and endorphin‐1 expression in the medullary cephalolateral–spinal pathway, reducing mechanical nociceptive sensitization and thus reducing pain. The E‐WAA analgesic treatment device added TENS on the basis of traditional WAA. Research by Zarei et al. showed that following high‐frequency TENS intervention, a specific area between the primary somatosensory cortex (SI) and the anterior cingulate cortex in the brain showed a significant increase in the brain network functional connectivity (FC) in the *γ* frequency range [39]. Acupuncture‐like TENS similarly modulated pain downstream conduction pathways [40], inhibiting pain signaling in the dorsal horn of the spinal cord and releasing enkephalins, endorphins, and endomorphins, activating opioid receptors, serotonin, *γ*‐aminobutyric acid (GABA), and muscarinic acid [41]. After the activation of opioid receptors, the G‐protein coupling mechanism causes changes in the ion channels and a decrease in the release of neurotransmitters such as acetylcholine and substance P from the nerve endings, thus blocking nociceptive conduction, raising the pain threshold, and producing an analgesic effect [42]. According to the above studies, the analgesic mechanism of the E‐WAA has been partially elucidated. These studies have partially elucidated the analgesic mechanism of the E‐WAA device. In the future, more in‐depth research will be conducted to further explore the device’s analgesic mechanisms.

The E‐WAA used in this study has four distinct advantages over conventional TENS. First, it can dynamically self‐adjust the accuracy of electrical stimulation by measuring human skin impedance, enhancing treatment efficacy and safety. Second, based on the theory of WAA, E‐WAA adds the concept of acupoint analgesia on top of TENS, which prolongs the curative effect. Third, the device is ergonomically designed for easy wearing on the wrist and ankle, allowing patients to adjust stimulation intensity and amplitude themselves, facilitating self‐management of treatment parameters. This makes it an ideal solution for managing pain in both medical and life scenarios. Finally, the use of E‐WAA reduces the psychological burden of patients during medical treatment, greatly reducing their stress levels. This study provides some reference for future clinical implementation.

## 5. Limitations

Due to the limited time, the 2‐week follow‐up may not reflect the long‐term effect of the intervention, and the relatively short treatment period of this study may affect the results. Additionally, strict inclusion criteria excluded certain patient groups, with significant differences from the baseline characteristics of the trial setup; for example, adolescents, pregnant women, elderly people over 70 years of age, and patients with other serious diseases were not included, limiting the generalizability of the results. Moreover, subgroup analyses (e.g., by disease severity or symptom duration) were not performed. Although the baseline characteristics were comparable between groups, the absence of subgroup exploration may overlook potential heterogeneity in treatment response.

Furthermore, the short follow‐up period limits the ability to fully evaluate the potential long‐term efficacy of E‐WAA. While this study provides preliminary evidence for its clinical effectiveness, additional studies with extended follow‐up are necessary to confirm sustained benefits. The study was conducted under controlled research conditions; thus, the applicability of the findings to broader real‐world community or nonspecialized clinical settings requires further validation.

Future studies will extend the treatment and follow‐up duration to assess long‐term efficacy and durability, further investigate the optimal frequency, intensity, and duration of electrical stimulation, and prespecify subgroup analyses to better understand factors influencing treatment outcomes. They will also explore the effectiveness and feasibility of E‐WAA in real‐world clinical practice to support broader clinical translation.

## Consent

Each participant provided written informed consent before enrollment.

## Conflicts of Interest

The authors declare no conflicts of interest.

## Author Contributions

Ping Shi​ and Fanfu Fang had full access to all of the data in the study and take responsibility for the integrity of the data and the accuracy of the data analysis.

Lele Huang, Wenjuan Song, Xiaowei Luan, and Lei Shi contributed equally to this work.

Concept and design: Ping Shi and Fanfu Fang.

Acquisition, analysis, or interpretation of data: all authors.

Drafting of the manuscript: Lele Huang, Wenjuan Song, Xiaowei Luan, Lei Shi, Shuangyue Li, Mengcheng Cai, Jiahao Du, Ping Shi, and Fanfu Fang.

Critical revision of the manuscript for important intellectual content: all authors.

Statistical analysis: Lele Huang, Wenjuan Song, Xiaowei Luan, Lei Shi, and Shuangyue Li.

Obtained funding: Shi and Fang.

Administrative, technical, or material support: Shuangyue Li, Mengcheng Cai, and Jiahao Du.

Supervision: Fanfu Fang.

## Funding

This work was supported by the National Key Research and Development Program of China (2019YFC1711800), “Yuan Hang” Medical Talent Program of Naval Medical University (2019‐YH‐06), Youth Science and Technology Talents Scheme ([2020]NQ06128), and Program of Shanghai Shenkang Hospital Development Center (SHDC2022CRD004).

## Supporting Information

The supplementary materials include the study protocol and the CONSORT 2010 Checklist.

## Supporting information


**Supporting Information** Additional supporting information can be found online in the Supporting Information section.

## Data Availability

The datasets produced and/or examined in the course of this study can be obtained upon reasonable request from the corresponding author.
