# Supplementary material for: Efficacy and Safety of Transcutaneous Acupoint Electrical Stimulation in the Treatment of Cervical Spondylosis: A Multicenter Randomized Open‐Labeled Controlled Clinical Study
Source: Pain Res Manag. 2025 Dec 5;2025:3949302. doi: 10.1155/prm/3949302 (PMC12767416; doi:10.1155/prm/3949302)
Supplement: Supplementary file 1 — Supporting Information Additional supporting information can be found online in the Supporting Information section. [file PRM-2025-3949302-s001.zip › Study_protocol.docx]

Efficacy and safety of electronic wrist-ankle acupuncture analgesia therapeutic device in the treatment of cervical spondylosis: a double-center, open-label, randomized controlled clinical study

School of Health Science and Engineering, University of Shanghai for Science and Technology, Shanghai

Department of Rehabilitation Medicine, The First Affiliated Hospital of the Naval Medical University, Shanghai

Department of Rehabilitation Medicine, Yueyang Hospital of Integrated Traditional Chinese and Western Medicine, Shanghai University of Traditional Chinese Medicine, Shanghai

Department of Rehabilitation Medicine, PLA Naval Medical Center, Shanghai

1. Background

Cervical spondylosis is a prevalent clinical condition that has become more common due to changes in work habits and intensity, resulting in an increase in people working with their heads down. The condition affects younger individuals more frequently and can be classified into various types, including nerve root, sympathetic, vertebral artery, cervical myelopathy, and mixed types. The most common symptoms are stiff neck, shoulder pain, and limited cervical flexion, with nerve root and cervical spondylopathy being the most frequent types.

Wrist and ankle acupuncture is a form of superficial skin acupuncture that has shown promising results in treating cervical spondylosis. However, it is invasive and poses a risk of infection, necessitating professional personnel to administer it. To address this, a research group has developed a novel wearable wrist and ankle electronic acupuncture analgesic treatment device that uses percutaneous electrical stimulation to mimic the effects of "wrist and ankle needle" treatment. This non-invasive treatment and management program is safe, dependable, and suitable for pain relief in all weather conditions.

The system has a range of treatment options, with adjustable amplitude and optional frequency to accommodate various types and levels of pain. It also has an efficient, stable, and low-power circuit design that ensures extended battery life and high safety. Personalized parameter settings based on biofeedback enable the electrical stimulation parameters to be tailored to the user's pain perception.

Cervical massage is another effective method for relaxing stiff neck muscles and restoring disarranged joints. When combined with the wearable wrist and ankle electronic acupuncture and analgesic therapy devices, it is possible to achieve a good therapeutic effect and improve treatment efficiency. This integrated approach can drive the development of related industries, such as the research and development of intelligent equipment for traditional Chinese medicine.

1. Study objectives

The objective of this study is to assess the effectiveness of a wearable electronic acupuncture and analgesic therapy device on the wrist and ankle, coupled with manipulation, in treating cervical spondylosis and nerve root-type cervical spondylosis. Furthermore, the study aims to compare the therapeutic effects of two different treatments for cervical spondylosis, namely, wearable wrist and ankle electronic acupuncture analgesic therapy instruments versus conventional wrist and ankle acupuncture.

The study will provide an impartial evaluation of the efficacy and safety of the wearable wrist and ankle electronic acupuncture and analgesic therapeutic instrument in clinical treatment of cervical spondylosis. It hopes to provide a more convenient and effective treatment method for cervical spondylosis patients and establish the foundation for future promotion of this treatment.

By combining traditional Chinese medicine theory with modern science and technology, this study endeavors to enhance the rehabilitation and treatment of patients with cervical spondylosis. The research is a significant step towards modernizing traditional Chinese medicine and offering patients with cervical spondylosis an improved treatment option.

1. Methods/design

A two-center, randomized, controlled, non-inferiority test clinical application research method was used to design the trial protocol.

1. Participants and recruitment
   1. Participants

A total of 164 patients with cervical spondylosis enrolled, at the departments of rehabilitation medicine of Yueyang Hospital of Integrated Traditional Chinese and Western Medicine of Shanghai University of Traditional Chinese Medicine and Changhai Hospital of Naval Medical University from June 2021 to December 2022, all of whom signed informed consent forms.

- 1. Sample design

According to previous clinical studies, the WAA group (*n*=30) had an effective rate of 86.0% after the intervention. The transcutaneous electrical stimulation group (*n*=30) had an effective rate of 83.3% after intervention. The sample size formula for this trial was as follows.

n = 2π( 1－π)[ $\frac{(\mu_{1 － \alpha}+ \mu_{1－ \beta})}{\delta}$]^2^

Where *n* is the sample size, α is the test level α = 0. 025 (one-sided), β is the type II error (β = 0.2), δ is the non-inferiority cutoff set at 0.2, the ratio of control group to experimental group was 1:1, and the dropout rate was 20%. Each group requires 82 patients, making a total of 164 patients needed.

4.3 Diagnostic criteria

(1). Typically, individuals over the age of 40 who have a prolonged history of using smartphones or other similar devices, as well as those who experience chronic strain, have a history of cervical degenerative diseases or trauma.

(2). Symptoms may consist of headaches, dizziness, localized neck stiffness, and tender points nearby. Movement of the neck may be limited.

(3). X-ray films may reveal pathological changes in the physiological curvature of the cervical spine, hyperplasia of the uncinate joint, narrowing of the vertebral space, and reduction of the intervertebral orifice. CT scans may show retrovertebral neoplasia and narrowing of the nerve root canal.

(4). Symptoms may include neck pain, radiating pain in the upper extremities, aggravation of neck extension, decreased sensation in the skin segment distribution of compressed nerve roots, abnormal tendon reflexes, muscular atrophy, decreased muscle strength, limited neck movement, positive tensile test, and head pressure test.

- 1. Inclusion criteria

(1) This study welcomes individuals of any gender between the ages of 20 and 70 years old.

(2) To be eligible, patients must meet the diagnostic criteria for cervical spondylosis, with noticeable pain symptoms and a VAS score of at least 4. Surgery is not recommended.

(3) Individuals who have not taken any analgesic medication in the two weeks prior to enrollment are eligible.

(4) All participants must provide informed consent and willingly volunteer to take part in this clinical trial.

- 1. Exclusion criteria

(1) Neck pain caused by other diseases.

(2) Patients with serious illnesses such as tumours, tuberculosis, ankylosing spondylitis, complicated with serious cardiovascular and cerebrovascular, liver, kidney, and other life-threatening primary diseases.

(3) People with cognitive, mental, or communication disabilities. Pregnant and lactating women should also avoid acupuncture massage.

(4) Local skin damage or other conditions not suitable for acupuncture massage.

(5) Participants who have participated in or are currently participating in other clinical trials within one month before enrollment should not undergo acupuncture massage.

4.6 Randomization and allocation concealment

This was a two-centre, open-label, analytically blinded, randomized controlled, non-inferiority clinical trial study. Patients were randomly divided into experimental and control groups in a ratio of 1:1 using a table of random numbers generated by SPSS 26.0 software, the randomized group numbers were sealed in light-proof envelopes, and when the subjects were accepted for the study, they were unsealed and enrolled in the right group.

1. Interventions
   1. Tuina

Referring to the "Rehabilitation Medicine in Chinese Medicine" textbook (Second Edition for the New Century) and the "Chinese Medicine Diagnosis and Treatment Program" (Department of Medical Affairs of the State Administration of Traditional Chinese Medicine), the following steps can be taken to perform neck massage:

(1). Use the roots of the palms of both hands to push and knead the lateral sulcus of the neck and the back along the direction of the trapezius muscle, the latissimus dorsi muscle and the sacrospinal fibers. Then use the rolling method to relax and loosen the tendons. The manipulation should be of moderate strength and should be continued for 3 minutes. As shown in the Fig.1.


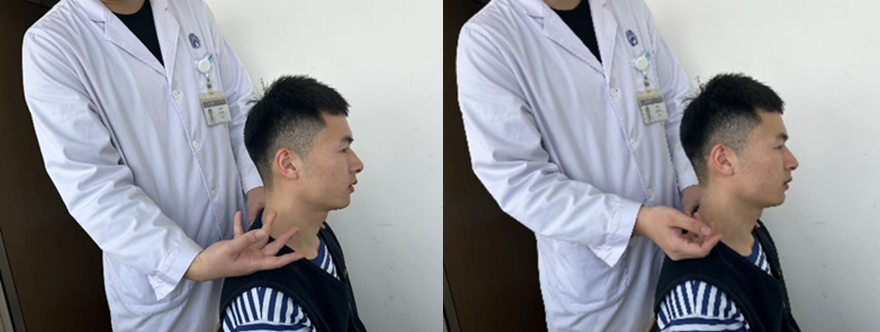


Fig.1 Massage relaxation

(2). Stand behind the patient and use the thumb and the remaining four fingers on both sides of the back of the neck. Knead evenly, up and down and back and forth for 5 minutes.

(3). After that, push the left thumb on the cervical spine (or transverse process), and use the right hand to hold the patient's chin. Ask the patient to relax the neck, bow the head, and flex the neck 15-30 degrees. Then ask the patient to follow the doctor's right hand in the state of flexion to the right and slowly turn the head. When the rotation reaches the maximum limit and meets resistance, the doctor should give a quick wrench with the right hand and push the top of the spinous process of the left hand thumb to the right to apply pressure. The coordinated action of the two hands should produce a "ka" sound of popping, which indicates the successful completion of the procedure. As shown in the Fig.2.


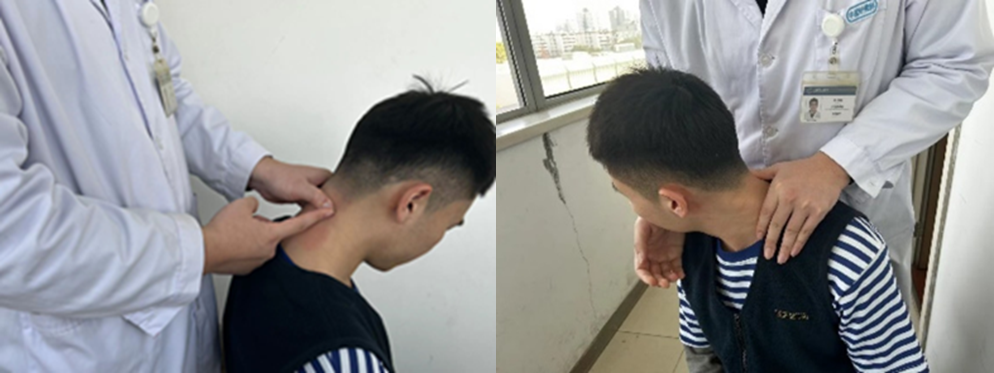

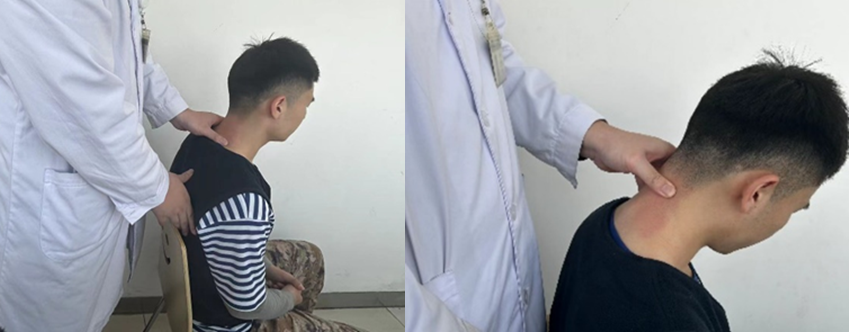


Fig.2 Massage pulling hand method

(4). Finally, pat the patient's back and scapula in a sitting or prone position. The operator should clench their fist or use a hollow palm to pat the patient's back and scapula with moderate strength, repeated 3-5 times, depending on the patient's comfort level. As shown in the Fig.3.


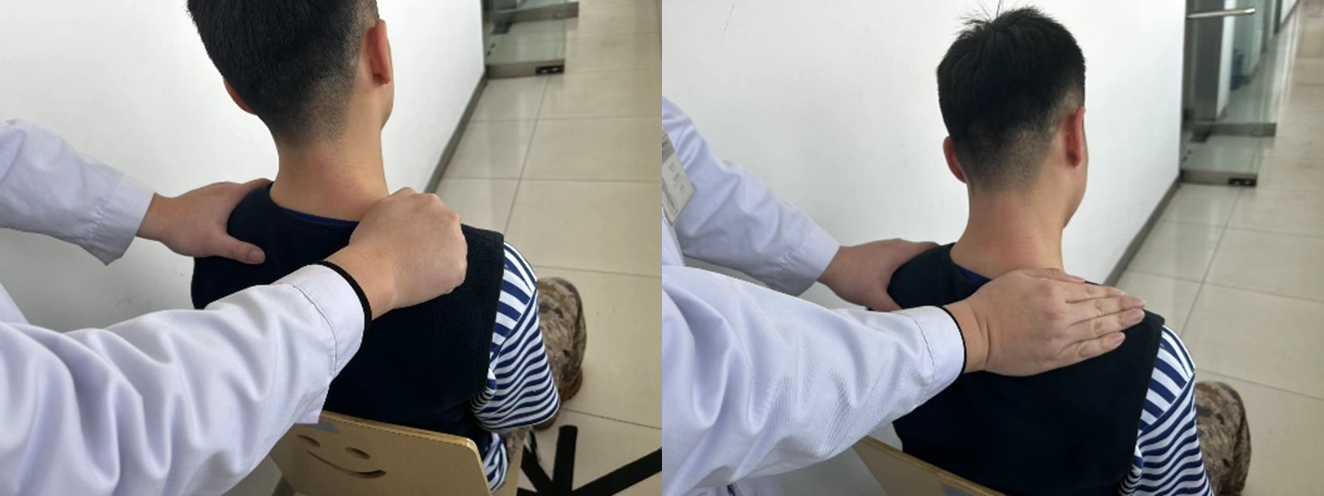


Fig.3 Relaxation after massage

5.2 Conventional wrist and ankle acupuncture treatment

Refering to the book "Wrist and Ankle Acupuncture" edited by Ling Changquan and published by Shanghai Science and Technology Press. The acupuncture points for this treatment are selected based on the location of the patient's cervical spine pressure pain point. To do this, you should choose the upper 5 or 6 zones of the wrist and ankle acupuncture treatment area.

5.2.1Location

Zone 5 represents the posterior temporal region, the posterior lateral part of the neck, the suprascapular fossa of the scapula gun, the scapular region, and the back and lumbar region that is vertically downward from the scapula.

Zone 6 represents the area on either side of the posterior midline of the trunk, opposite the anterior zone 1. This includes the posterior head, occiput, posterior cervical region, spine, cervical spinous processes to the trapezius margin, thoracic spinous processes to the medial margin of the scapula, sides of the sacral median ridge to the coccyx, and anus.

To locate the acupuncture points for the treatment, you should look at the upper 5 zone which is in the middle of the dorsal wrist, between the two edges of the radius and ulna. The upper 6 zone is located on the dorsal side of the wrist, 1cm from the ulnar margin on the side of the little finger.

5.2.2 Operation

The treatment area of the patient's wrist was sterilized, specifically the upper 5 and upper 6 zones. Acupuncture was performed using sterile acupuncture needles that measured 0.25 mm × 25 mm. The operator held the lower part of the needling point with one hand and grasped the handle of the acupuncture needle with the thumb, index finger and middle finger of the other hand. As shown in the Fig.4.


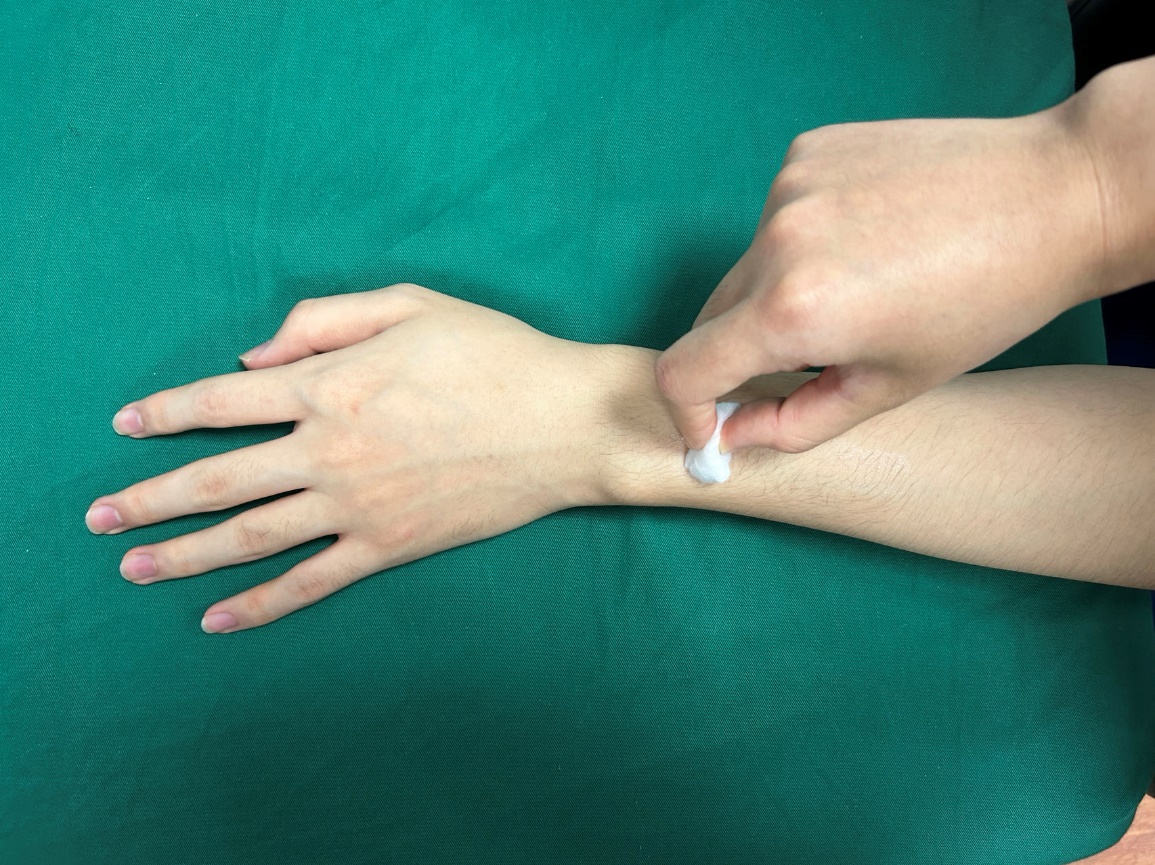


Fig.4 Sterilize the injection site

The operator then inserted the therapeutic needles into the patient's superficial subcutaneous tissues at a 30° angle to the skin, ensuring that the body of the needles felt loose under the skin. As shown in the Fig.5.


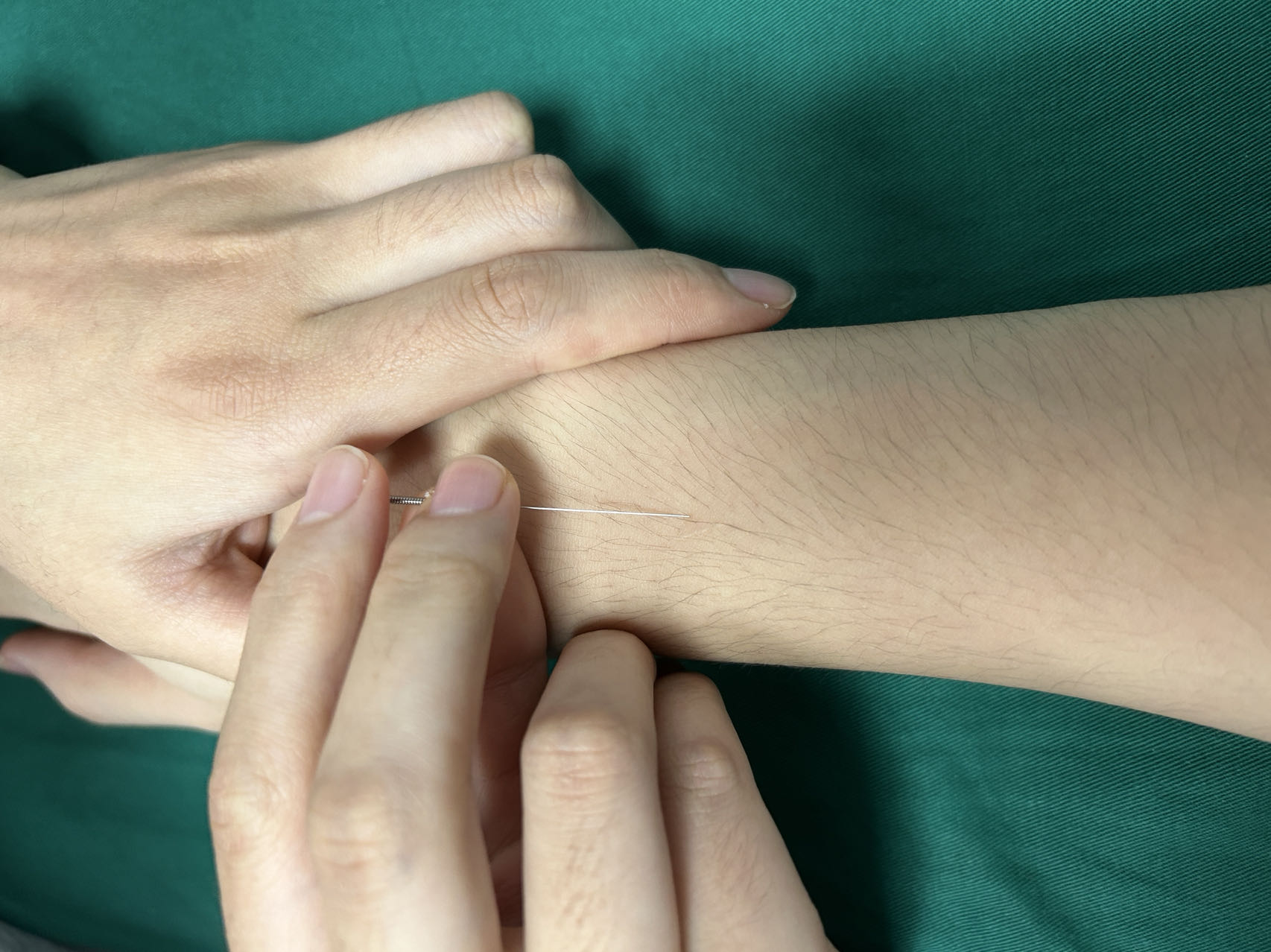


Fig.5 Wrist and ankle acupuncture process

The patient did not experience any acidity, numbness, distension, or pain during the treatment process. The body of the needle naturally hung down close to the skin surface. After the needling was completed, the operator covered the entrance of the needle with a medical sterile dressing, used medical tape to fix the handle of the needle, and left the needle in place for 30 minutes. As shown in the Fig.6.


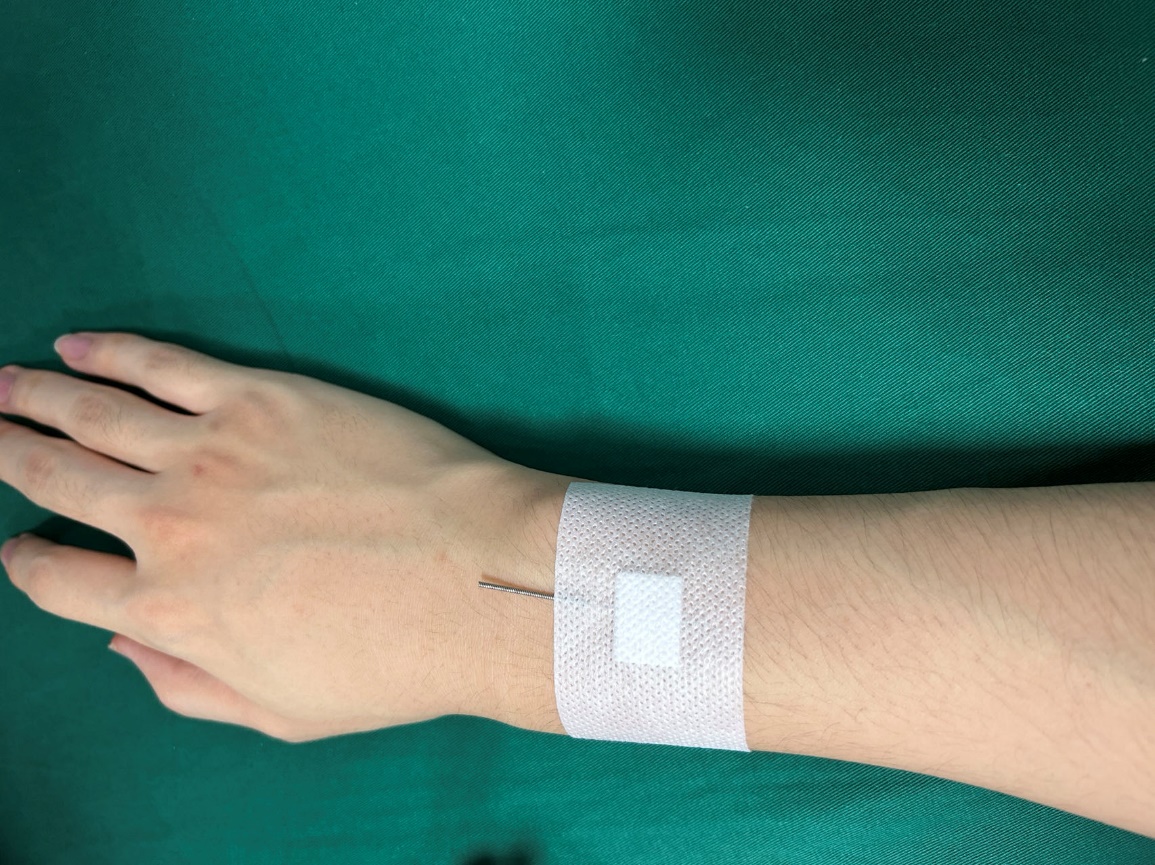


Fig.6 Keep the wrist and ankle needle

The course of treatment was one session every other day, for a total of 6 sessions over a period of 12 days.

- 1. E-WAA

The upper 5 or 6 zones of the wrist and ankle acupuncture treatment area are selected according to the location of the patient's cervical spine pressure points. Zone 5 represents the two sides of the spine; Zone 6 represents the spinal region.

5.3.1 Location

Upper 5 zone (in the center of the dorsal wrist, between the two edges of the radius and ulna)

Upper zone 6 (on the dorsal side of the wrist, 1 cm from the ulnar margin on the side of the little finger)

5.3.2 Operation

Use the E-WAA developed by the research group. Expose the skin of the patient's treatment area and sterilize it during treatment. As shown in the Fig.7.


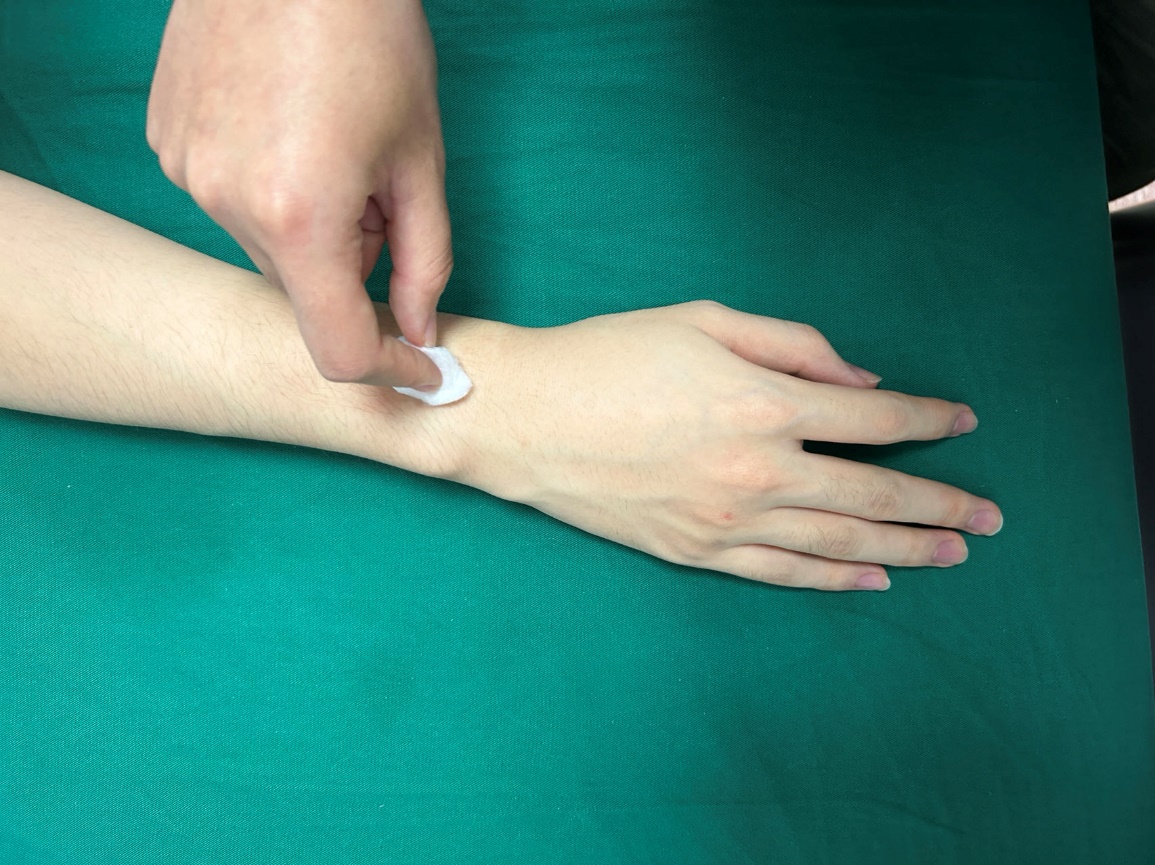


Fig.7 E-WAA disinfection

Place two transcutaneous electrical stimulation probes (electrodes) on the upper 5 or upper 6 zones of the wrist and ankle acupuncture point, adopt the upper and lower juxtaposition method, and the electrodes are close to the skin. As shown in the Fig.8.


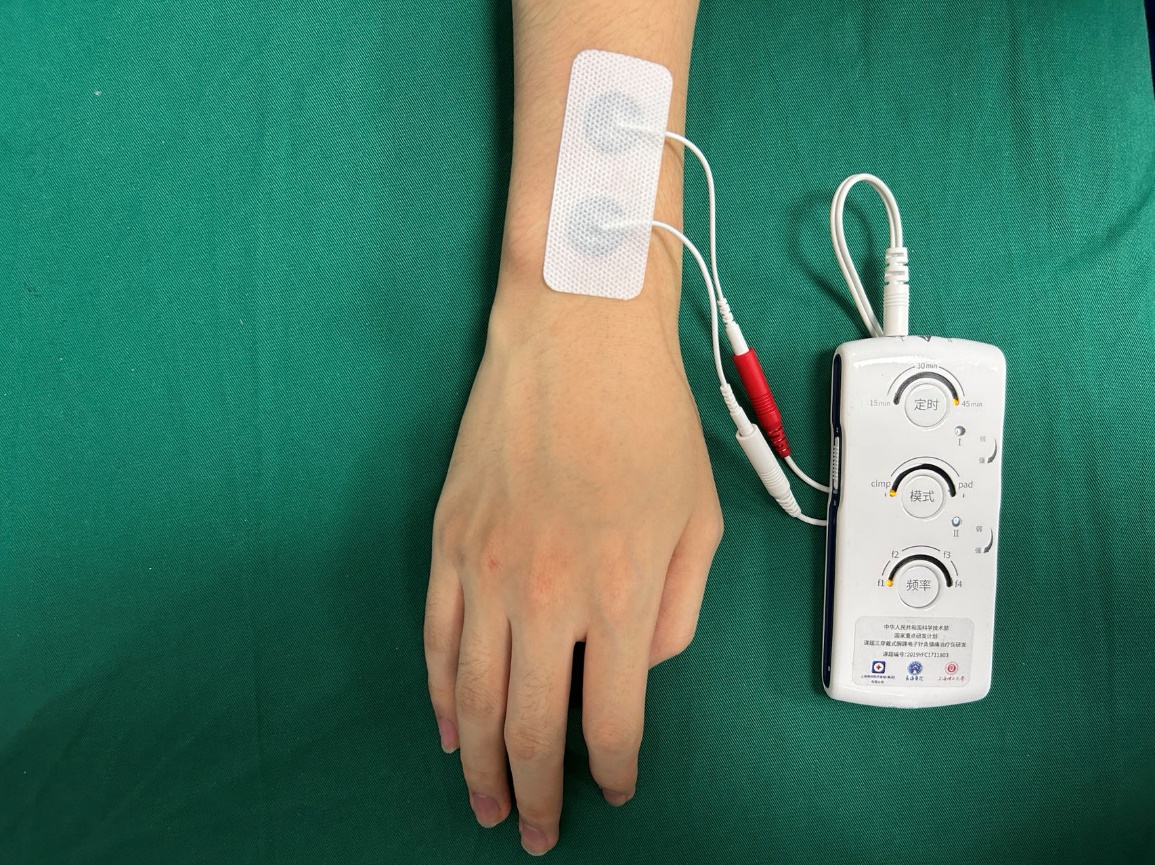


Fig.8 The electrode patch is attached to the skin

Select the patient's appropriate pulse parameters, select the f1 waveform and comfortable intensity, stimulation duration 30min, the end of the treatment, turn off the machine. As shown in the Fig.9.


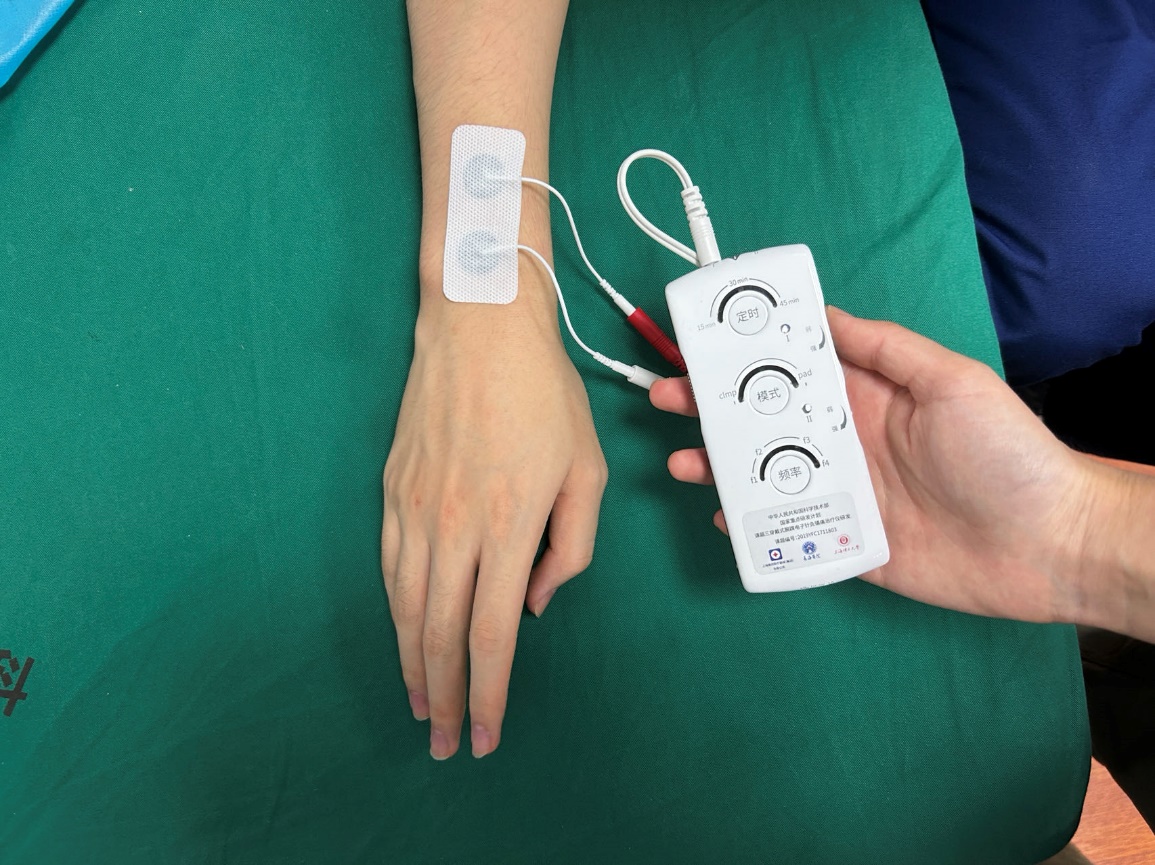


Fig.9 Adjust therapeutic instrument parameters

Course of treatment 1 time every other day, a total of 6 times of treatment, a total of 12 days.

1. Outcome measurements

6.1 Main outcome

Effective rate at the end of 6 treatments

The clinical efficacy was assessed concerning the clinical efficacy evaluation criteria of the Chinese medicine industry standard of the People's Republic of China - Chinese medicine disease diagnosis efficacy standard (ZY/T001.1-94), as well as the improvement rate of the VAS score. The improvement rate of the VAS score = [(pre-treatment VAS score - post-treatment VAS score) ÷ pre-treatment VAS score] × 100%. VAS score improvement rate = [(pre-treatment VAS score - post-treatment VAS score) ÷ pre-treatment VAS score] × 100%.

Cure: complete disappearance of symptoms, no localized pain, restoration of cervical spine function, VAS score of 0;

Significantly effective: significant relief of symptoms, significant improvement of localized pain, significant improvement of cervical spine function, VAS score improvement rate >60%;

Effective: symptoms improved, local pain relieved, cervical spine function relieved, VAS score improvement rate of 30%~60%;

Ineffective: no improvement in symptoms and signs, VAS score <30%.

6.2. Secondary outcome

Neck Disability Index (NDI).

Cervical Range of Motion (CROM)

The Likert Score to evaluate the satisfaction level of doctors and patients using the equipment developed in this project.

6.3 Safety Indicators

The patients' heart rate, blood pressure, respiration and pulse were closely observed before and after the treatment and during the treatment. Record the possible adverse reactions in the course of treatment in a truthful and detailed manner, and evaluate the incidence of adverse (serious) events between groups and the incidence of device-related (serious) adverse events.

Safety evaluation grading:

Grade 1: No adverse reactions;

Grade 2: mild adverse reactions, can continue clinical treatment without corresponding treatment;

Grade 3: moderate adverse reactions, patients can continue to receive treatment after requiring appropriate treatment;

Grade 4: serious adverse reactions and discontinue the trial.

1. Ethical considerations

The data used in this study were from patients with cervical spondylosis. The data collected in this study are for the purpose of this study only. Enrolled patients are required to sign an informed consent form, and patient information in the forms and pictures will not be recognized in this trial. Ethical approval (Anonymized) for this study was obtained from the Ethics Committee of Anonymized. Meanwhile, it has been registered in the Clinical Trial Registration Center (Anonymized).
